# Continuous Wavelet Transform Analysis of Acceleration Signals Measured from a Wave Buoy

**DOI:** 10.3390/s130810908

**Published:** 2013-08-19

**Authors:** Laurence Zsu-Hsin Chuang, Li-Chung Wu, Jong-Hao Wang

**Affiliations:** 1 Institute of Ocean Technology and Marine Affairs, National Cheng Kung University, 1, Da-Hsueh Rd., Tainan 70101, Taiwan; E-Mail: zsuhsin@mail.ncku.edu.tw; 2 Coastal Ocean Monitoring Center, National Cheng Kung University, 1, Da-Hsueh Rd., Tainan 70101, Taiwan; 3 Water Resources Agency, Ministry of Economic Affairs, 9-12F, 41-3 Sec.3 Hsin-Yi Rd., Taipei 10651, Taiwan; E-Mail: coa1234s@yahoo.com.tw

**Keywords:** wave accelerations, wavelet spectrum, sea surface elevations, wavelet transform

## Abstract

Accelerometers, which can be installed inside a floating platform on the sea, are among the most commonly used sensors for operational ocean wave measurements. To examine the non-stationary features of ocean waves, this study was conducted to derive a wavelet spectrum of ocean waves and to synthesize sea surface elevations from vertical acceleration signals of a wave buoy through the continuous wavelet transform theory. The short-time wave features can be revealed by simultaneously examining the wavelet spectrum and the synthetic sea surface elevations. The *in situ* wave signals were applied to verify the practicality of the wavelet-based algorithm. We confirm that the spectral leakage and the noise at very-low-frequency bins influenced the accuracies of the estimated wavelet spectrum and the synthetic sea surface elevations. The appropriate thresholds of these two factors were explored. To study the short-time wave features from the wave records, the acceleration signals recorded from an accelerometer inside a discus wave buoy are analysed. The results from the wavelet spectrum show the evidence of short-time nonlinear wave events. Our study also reveals that more surface profiles with higher vertical asymmetry can be found from short-time nonlinear wave with stronger harmonic spectral peak. Finally, we conclude that the algorithms of continuous wavelet transform are practical for revealing the short-time wave features of the buoy acceleration signals.

## 1. Introduction

Wind-generated gravity waves are among the most significant phenomena on the ocean. However, the mechanics and features of these types of waves are highly complex and random because of the combined influences of meteorological, hydrological, oceanographic and topographical factors. Studies of wind-generated gravity waves have continued ever since their contributions to water wave mechanics were recognised more than a century ago. To increase our practical knowledge of wind-generated gravity waves field measurements must be performed, but most measurement sensors are only suitable for use nearshore or in shallow water areas. Apart from remote sensing devices, moored buoys and vessels are the only platforms suitable for wave measurement in deep water areas [1]. Data buoys have been the most popular means of operational wave monitoring since the 1970s [2].

A wave buoy floats on the sea surface and moves up and down with the waves. Different sensors, such as GPS and accelerometers, have been developed and installed on buoys for measuring the waves. Although some studies have proved the practicability of GPS sensors for measuring waves [3,4], the accelerometer is still the most popular sensor for wave measurement by wave buoys. An accelerometer placed inside a buoy can measure its tri-axial acceleration. In characterising the wave features, the first step is often to analyse the heave (vertical) motion of the sea surface. Assuming that the buoy has perfect wave-following characteristics or using the appropriate response factors for the buoy measurement system [5,6], we can obtain the sea surface vertical accelerations from the accelerometer inside the buoy. In theory, the water surface displacement or the heave motion data can be estimated by a double integration of the heave acceleration time series. Although the concept is simple, there are difficulties in its successful implementation. In each integration low frequency components of the signal are amplified and high frequency components are reduced, and the phase for each frequency is changed, too. Thus, any offset of the acceleration signal will dominate the results of calculated displacements and as a result, the heave data may drift over time. Arraigada and Partl [7] demonstrated the effect of double integration of a periodic waveform using a numerical example. According to their result, even a little constant offset error in the measured accelerations may produce a quadratic baseline error in calculated displacements. To overcome this issue, we often need to use a high-pass filter with a proper cut-off frequency. In contrast with the double integration of vertical accelerations, another method is to directly address the acceleration signals in the spectral domain. The wave acceleration records obtained from the accelerometer measurements can be transformed into an acceleration spectrum using a suitable spectral transformation algorithm. The wave spectrum of sea surface elevations, which is also known as the variance density spectrum [8], can be obtained from the acceleration spectrum using a transfer function [9,10]. Tucker [11] applied the first-order wave theory and proved that the transfer function between the spectrum of the acceleration signal and the wave spectrum only depends on the wave frequencies.

To obtain a spectrum from acceleration records, a Fourier transform of the spectral transform has often been used in the past. The theory of the Fourier transform assumes the signal is stationary. As a result, the wave spectrum estimated by Fourier transform provides enough information to describe the sea-surface elevations as a stationary and Gaussian process. However, in Nature, most real signals are non-stationary, as are wave signals. Liu [12] showed that the time series of wind waves are not at all comparable to random Gaussian signals, which should be stationary, even for segment lengths of 5 min. To understand instantaneous wave features from a set of wave records, we must implement a method which is practical for non-stationary signal analysis.

The application of the continuous wavelet transform has become increasingly common since its inception in the early 1980s. Compared to the Fourier transform which is based on the concept of frequency, the continuous wavelet transform is based on the concept of time-frequency localization. Wavelet transforms are capable of obtaining orthonormal basis expansions of signals using time-frequency atoms that enable us to localize the signals in time and frequency domains. Due to the feature of time-frequency localization, the wavelet theory has been applied successfully to solve various geophysical problems [13]. The wavelet transform is now recognised as a useful, flexible, and efficient technique to analyse non-stationary signals and wave records that are obtained from laboratory experiments or field observations too. Some studies have used the wavelet spectrum of sea elevations, which is defined as a local time-frequency energy density by the wavelet transform [14], to identify groups of waves and breaking waves that occur at different times [15,16]. Massel [17] examined the energy growth during the wave generation stage through a wavelet algorithm. In addition to the wavelet spectrum, the instantaneous or short-time features of the sea surface elevations remain quite significant for some important topics such as the studies of wave grouping and freak waves [15,18]. The reconstruction of signals using the inverse wavelet formula was then proved in the 1960s [14].

Up to now, most of the related studies on wavelet spectrum have been applied to sea elevation signals. However, most of the *in situ* wave data were measured by accelerometer-equipped buoys. If the wavelet spectrum of ocean waves and sea surface elevations can be estimated from acceleration signals, we can explore more non-stationary and even nonlinearity characteristics of ocean waves. For this reason, we aim to develop a complete procedure of wavelet-based algorithm which is capable of obtaining a wavelet spectrum and sea surface elevations from buoy acceleration records. Similar to the Fourier-based algorithm procedure, a transfer function is also necessary to convert the wavelet spectrum of acceleration signals into the wavelet spectrum of sea surface elevations. The transfer function from Tucker [11] is valid under this assumption that any wave system can be represented by the sum of infinitely extending sinusoidal wave trains. However, this transfer function is invalid for the wavelet transform if the second derivative of a wavelet function cannot retain the shape of the original function. In this study, we used the Morlet wavelets instead, because the Morlet wavelets are Gaussian-modulated sine/cosine functions. The transfer function from Tucker [11] is then approximately valid when the Morlet wavelet function is applied to it. In a later section, we verify the feasibility of the wavelet-based algorithm based on using the Morlet wavelet function. Finally we apply the algorithm to extract the short-time wave features from the wavelet spectrum and the synthetic sea surface elevations of observational acceleration signals, so as to confirm the significance of the wavelet-based algorithm on our issues.

## 2. Theoretical Preliminaries

Based on the continuous wavelet transform (CWT) theory, the acceleration signal can be broken into various wavelets that are scaled and shifted versions of a pre-chosen mother wavelet function. The acceleration signal *A_c_*(*t*) is a time series of accelerations. The continuous wavelet transform of acceleration signal for a transformed wavelet function *ψ_b_*_,_*_a_* is as follows:

(1) $$W_{Ac}(b,a)=\langle \psi_{b,a}|A_{c}(t)\rangle ,$$

where the scale parameter *a* is related to the dilated frequency in the time domain. This parameter is a normalisation that gives all dilated versions of the mother wavelet the same energy. That is, the scale parameter is the ratio of the size of the dilated wavelet to the size of the mother wavelet. The translation parameter *b* corresponds to the position of the wavelet as it shifts through the time domain. |*W_AC_*(*b,a*)|^2^, derived from Equation (1), is referred to as the scalogram [19,20], which presents a local time-scale energy density. Equation (1) can also be expressed as follows:

(2) $$W_{Ac}(b,a)=a^{-1/2}\int \psi_{b,a}^{*}(t)A_{c}(t)dt$$

(3) $$\psi_{b,a}(t)=\frac{1}{\sqrt{a}}\psi \left(\frac{t-b}{a}\right),$$

where ψ*_b__,_*_a_*(*t*) is the complex conjugate of the wavelet function *ψ_b_*_,_*_a_*(*t*). The mother wavelet function *ψ* must satisfy the following admissibility condition:

(4) $$C_{\psi }=(2\pi )\int_{-\infty }^{\infty }\frac{{\left|\psi^{\prime }(\omega )\right|}^{2}}{\left|\omega \right|}d\omega <\infty$$

In most cases, this condition may be reduced to the (only slightly weaker) requirement that *ψ* has a zero mean [21]:

(5) $$\int_{-\infty }^{\infty }\psi (t)dt=0$$

The relationship between the wavelet function and the mother wavelet function in the Fourier (spectral) space can be expressed as follows:

(6) $${\psi^{\prime }}_{b,a}(\omega )=\sqrt{a}\exp(-ib\omega )\hat{\psi }(a\omega ),$$

where *ψ′* is the Fourier space of function *ψ*, which represents the function in the spectral space. *ω* is the angular frequency. *W_AC_*(*b,a*) conserves the norm of the signal; thus its total energy can be expressed as follows [22]:

(7) $$E_{n}={\int |A_{c}(t)|}^{2}dt={\iint |W_{Ac}(b,a)|}^{2}\frac{\text{dadb}}{a^{2}}$$

To implement Equation (2), it is necessary to first choose a mother wavelet function *ψ*. The Morlet wavelet function, which is a wavelet function commonly used in many applications, is chosen here for extracting the wave information from the acceleration signal. The Morlet mother wavelet function and its function in the Fourier (spectral) space, as defined in Equations (8) and (9), were used throughout the implementation procedures in this study:

(8) $$\psi (t)=\exp(i\omega_{0}t)\exp(-0.5t^{2})$$

(9) $$\psi^{\prime }(\omega )={\left(2\pi \right)}^{-0.5}\exp(-0.5{\left(\omega -\omega_{0}\right)}^{2})$$

In these equations, *ω*_0_ is a constant that forces the admissibility condition, as shown in Equation (4), to be satisfied. A value of 5.5 was suggested for this constant in a study by [23]. Equation (6) shows that the angular frequency is transformed from *ω* into *aω* after scaling and shifting a mother wavelet function *ψ′*(*ω*) to a wavelet function *ψ′_a_*(*ω*). As shown in Equation (9), *ω*_0_ is also the peak frequency of the mother Morlet function in the frequency domain. After transformation, the new peak frequency of the Morlet wavelet function becomes *ω*. The relationship between *ω*_0_ and *ω* is given as follows [22]:

(10) $$\omega =\omega_{0}/a$$

Equation (10) shows that we can obtain *ω* from the scale parameter *a*. The translation parameter *b* from the CWT theory denotes the translation distance of the wavelet function from the original position of the signal. In other words, the translation parameter *b* indicates the position (time) of the signal. As a result, the function |*W_AC_*(*b,a*)|^2^ can be expressed as |*W_AC_*(*t,ω*)|^2^, which represents the spectral information at different positions (times) *t*. In addition to |*W_AC_*(*t,ω*)|^2^, the spectral information of the sea surface elevations |*W_η_*(*t,ω*)|^2^ can be obtained using the equations discussed above if the acceleration signal *A_c_*(*t*) are replaced by the sea surface elevation signal *η*(*t*).

To estimate the time series of the sea surface elevations *η*(*t*) from the scalogram, the inverse continuous wavelet transform (ICWT) theory is applied:

(11) $$\eta (t)=\frac{1}{C\psi }\int_{0}^{\infty }\int_{-\infty }^{\infty }W_{\eta }(b,a)\psi_{b,a}(t)db\frac{da}{a^{2}},$$

where |*W_η_*(*b,a*)|^2^ is the wavelet scalogram of the sea surface elevations, which is related to |*W_AC_*(*b,a*)|^2^. Based on the theory discussed above, the wavelet scalogram of the acceleration signal |*W_AC_*(*b,a*)|^2^ can be estimated. To implement Equation (11), we first need to obtain the wavelet scalogram of the sea surface elevations |*W_η_*(*b,a*)|^2^. Tucker [11] derived the relationship between the spectrum of the acceleration signal |*W_AC_*(*ω*)|^2^ and the spectrum of the sea surface elevations |*W_η_*(*ω*)|^2^:

(12) $${\left|W_{\eta }(\omega )\right|}^{2}=\omega^{-4}{\left|W_{Ac}(\omega )\right|}^{2}$$

Because the Morlet wavelets are Gaussian-modulated sine/cosine functions, Equation (12) is approximately valid using the Morlet wavelet function. In the later section, we will verify the feasibility of the wavelet-based algorithm while using the Morlet wavelet function.

As discussed above, we can transform the scalogram of acceleration signal |*W_AC_*(*b,a*)|^2^ into the wavelet spectrum |*W_AC_*(*t,ω*)|^2^ based on Equation (10). The wavelet spectrum of accelerations can now be applied to Equation (13) to obtain the wavelet spectrum of sea surface elevations |*W_η_*(*t,ω*)|^2^ which represents the frequency spectra at different time *t*:

(13) $${\left|W_{\eta }(t,\omega )\right|}^{2}=\omega^{-4}{\left|W_{Ac}(t,\omega )\right|}^{2}$$

Then we can transform |*W_η_*(*t,ω*)|^2^ into a scalogram of sea surface elevations |*W_η_*(*b,a*)|^2^. Finally we can synthesize the sea surface elevations *η*(*t*) from |*W_η_*(*b,a*)|^2^ through Equation (11).

## 3. Analysis of Wave Data

**Figure 1.**
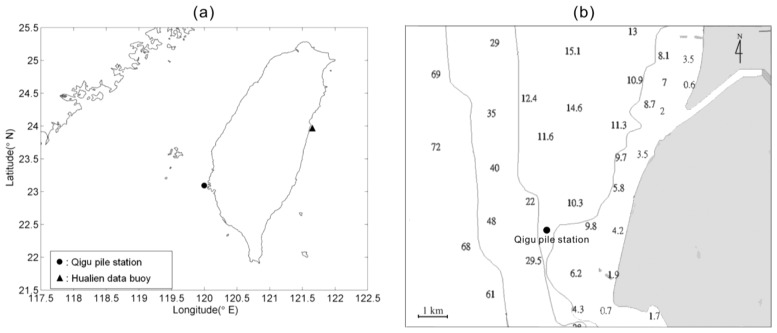
The location and surrounding bathymetry of the Qigu pile station. The numbers in Figure 1b stand for water depths. The pile station is located approximately 3 km from the western coast of Taiwan, where the water depth is 15 m.

To verify the numerical accuracy of computing Equation (13), the estimated wavelet spectrum of sea surface elevations, which is derived from the wavelet spectrum of wave accelerations by means of Equation (13), should be compared to the wavelet spectrum of observational sea surface elevations. Accordingly, an *in situ* dataset of sea surface elevations and accelerations for the same measurement location and duration was required to conduct an inspection. However, it is quite difficult to collect sea surface elevations data from a floating platform. Although a GPS sensor could be used to obtain the sea surface displacement records, we do not have the simultaneous observational records in our study site. To address this issue, the sea surface elevation records, measured by ultrasonic wave gages on a pile station, were chosen to develop the corresponding synthetic acceleration data by means of double forward difference. The location and the surrounding bathymetry of the pile station are shown in Figure 1. A total of 1,500 time-series records of sea surface elevations, collected from the pile station, were used to assess the accuracies of the wavelet spectra and the synthetic sea surface elevation signals which are estimated from the wavelet theory. The significant wave height and the mean wave period conditions of most of the data records are 0.5–2 m and 4–6 s, respectively. These wave cases are recorded from August 2000 to January 2001. The waves during this duration are often higher because of the influences of typhoons and winter monsoon. The wave height and period were calculated based on the zero-up-crossing method [24]. The sampling rate of these records is 2 Hz. For the case in each hour, the duration of data acquisition is 10 minutes, which indicates 1,200 data points will be recorded. To apply the fast Fourier transform algorithm to our study, we selected the first 1,024 data points from each case for further analysis.

The estimated wavelet spectrum |*W_η_*(*t,ω*)|^2^, calculated by the Equation (13), will be very strong at very-low-frequency bins because of the influence of *ω*^−4^ on |*W_AC_*(*ω*)|^2^. The very-low-frequency bins mentioned in this paper are the frequency bins that are lower than the common frequency bins of wind-generated waves. Many studies considers this energy density in the very-low-frequency band as noise [25]. In this paper, a high-pass filter with a rectangular window, for which the cut-off frequency was set at 0.05 Hz, was used to eliminate the low-frequency noise. The reason is that our study mainly focused on the wind-generated waves with typical periods of 2–20 s. Compared to wind-generated waves, the longer waves (such as infra-gravity waves and surges) have much weaker amplitudes. Hence, we ignore the Gibbs effect [26] while reconstructing the wind-generated waves.

To verify the accuracy of estimated wavelet spectra of sea surface elevations, which are derived from Equation (13), we first chose a dataset of wave height 0.78 m and mean wave period 3.9 s at the Qigu pile station and compared the wavelet spectrum of observational sea surface elevations with its estimated wavelet spectrum of sea surface elevations. The so called estimated wavelet spectrum of sea surface elevations was calculated from the wavelet spectrum of synthetic wave accelerations by Equation (13). Also, the synthetic wave accelerations were estimated from the double forward difference of observational sea surface elevations. Since the wavelet spectrum is a measure of the energy distribution over time and frequency of the wave data, the energy densities of wavelet spectrum at higher frequencies are well localized in time. Besides, the uncertainty in frequency localization increases as the frequency increases. Consequently, the energy densities at the higher frequencies are more scattered in frequency domain than those at the lower frequencies. This is known as the Heisenberg's Uncertainty Principle [17].

**Figure 2.**
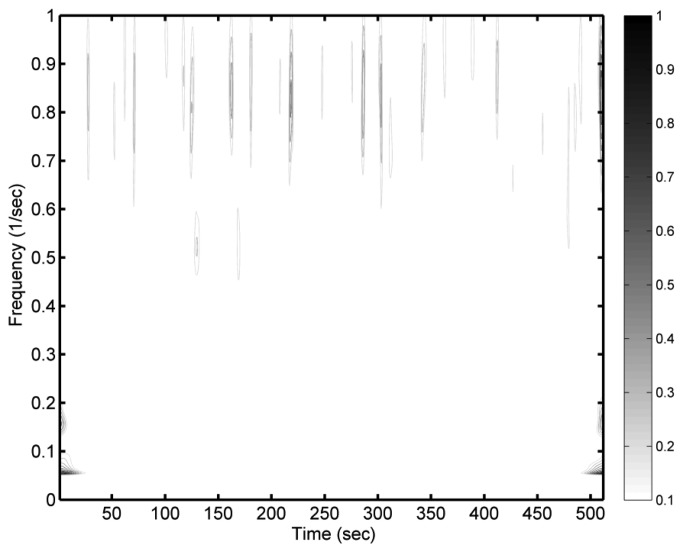
The normalised differences between two wavelet spectra of sea surface elevations. One was directly calculated from an observational sea surface elevation record, and the other wavelet spectrum was estimated from the synthetic acceleration data which was derived from the same observational record of sea surface elevations by means of double forward difference. To estimate the wave spectrum of sea surface elevations from the synthetic acceleration data, the transfer function in Equation (13) was applied here. The normalised difference was their differences of two wavelet spectra divided by the maximum difference value.

Figure 2 shows the normalised differences between a wavelet spectrum of observational sea surface elevations and an estimated wavelet spectrum of sea surface elevations, which was derived from synthetic wave acceleration data. The differences between the two wavelet spectra in the low-frequency bins are quite obvious, especially at the beginning and the end of the time considered. Those noticeable differences are caused by the effect of spectral leakage, which is also called the “cone of influence” in many literatures [14]. Huang *et al.* [27] noted that the leakage of the Morlet wavelet is generated by the limited length of the basic wavelet function, which makes it difficult to quantitatively define the energy–frequency–time distribution. Compared to the differences in the low-frequency bins, higher-frequency bins (>0.7 Hz) have weaker differences. Because we synthesized the acceleration data from the sea surface elevation records using the double forward difference, the high-frequency signals are more sensitive than the low-frequency signals while preceding the double forward difference. As a result, the weaker differences between two wavelet spectra in higher-frequency bins can be observed.

In Figure 2 we used the normalised difference to demonstrate the possible effects of applying the Equation (13) in estimating the wavelet spectra of sea surface elevations from a wavelet spectrum of synthetic acceleration signal. To further verify the accuracies of estimated wavelet spectra of sea surface elevations, which are derived from Equation (13), we adopted 1,500 wave records of sea surface elevations from the pile station to imitate their corresponding synthetic accelerations and then estimate their wavelet spectra of accelerations. The correlation coefficients *C_c_* could now be defined as Equation (14) to measure the accuracy of our approach:

(14) $$C_{c}=\frac{E\lfloor ({\left|x(t,\omega )\right|}^{2}-\mu_{x})({\left|y(t,\omega )\right|}^{2}-\mu_{y})\rfloor }{\sigma_{x}\sigma_{y}}$$

where |*x*(*t,ω*)|^2^ are wavelet spectra of 1,500 time-series records of observational sea surface elevations, and |*y*(*t,ω*)|^2^ are estimated wavelet spectra of sea surface elevations derived from 1,500 sets of synthetic acceleration data. Note that the synthetic wave acceleration data were derived from the observational sea surface elevations records by double forward difference. To convert the wavelet spectra of synthetic accelerations into wavelet spectra of sea surface elevations |*y*(*t,ω*)|^2^, the transfer function in Equation (13) was applied here. *μ_x_* and *μ_y_* are the mean values of |*x*(*t,ω*)|^2^ and |*y*(*t,ω*)|^2^, respectively; *σ_x_* and *σ_y_* are the standard deviations of |*x*(*t,ω*)|^2^ and |*y*(*t,ω*)|^2^, respectively.

**Figure 3.**
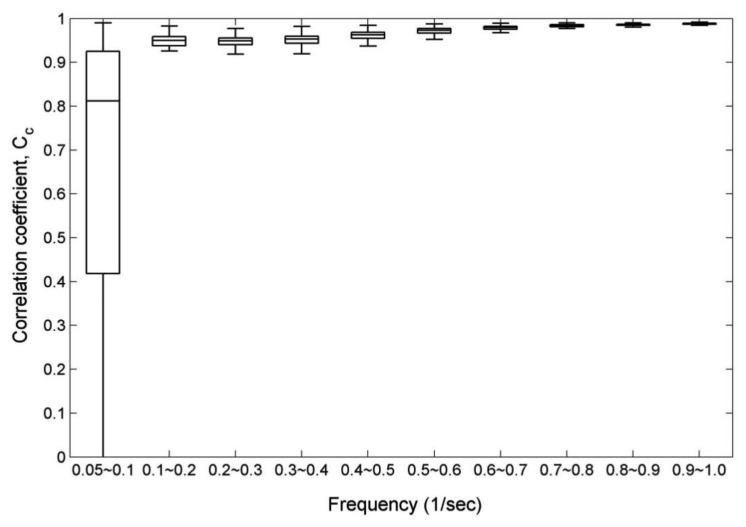
A box-whisker plot of correlation coefficients for paired wavelet spectra of 1,500 sea surface elevation records. One set of wavelet spectra was directly calculated from observational sea surface elevation records, and the other set was estimated from the synthetic acceleration data which were derived from observational records of sea surface elevations by means of double forward difference. The top and bottom of each box are the 25th and 75th percentiles of the samples, respectively. The line in the middle of each box is the sample median. The upper and lower whiskers present the highest and lowest results from the samples.

The box-whisker plot in Figure 3 was used to present the correlation coefficients of 1,500 wave data set for each frequency bin. The noises in the very-low-frequency bins are strong, but we only discuss the energy in the frequency bands that are greater than 0.05 Hz as mentioned previously. Figure 3 reveals that the correlations between the two wavelet spectra are greater in higher-frequency bins. Because of the transfer function *ω*^−4^ in Equation (13), the energy densities in lower frequency bins are substantially amplified. Especially, the correlations in the low-frequency bins of 0.05–0.1 Hz are poor and unstable although the cut-off frequency has been applied to the wavelet spectrum. We might set a higher value for the cut-off frequency, but upon doing so parts of wind-generated wave energy density could be eliminated. In addition to the noise in very-low-frequency bins, the observational acceleration signals of buoys also include some electronic noise and, as it is sampled, digitization noise. The frequency dependent noise correction function was intended to compensate for small levels of electronic and digitization noise that might appear in acceleration spectra [28,29]. However, the noises caused by the transfer function *ω*^−4^ at very-low-frequency bins are more evident than electronic noise when the wavelet spectrum is estimated by Equation (13). In a later section, we will focus on the influences of different cut-off frequency thresholds on the accuracy of the estimated wavelet spectrum.

**Figure 4.**
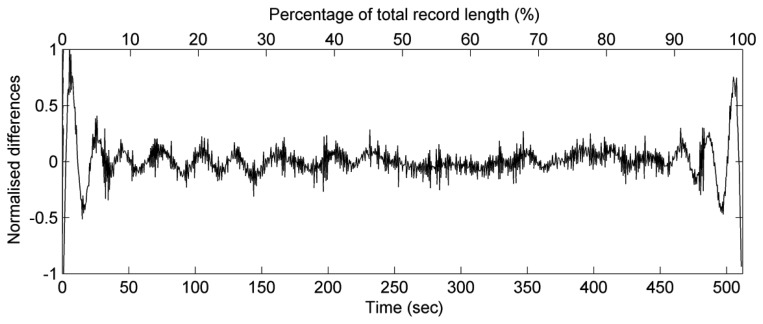
The normalised differences between the synthetic sea surface elevations and the observational sea surface elevations.

Since the estimated wavelet spectra of sea surface elevation records have be derived, the synthesized data sets of sea surface elevations can be also imitated through the inverse CWT algorithm of the estimated scalogram of the sea surface elevation record. It is necessary to measure the accuracies of synthetic sea surface elevation records. To obtain the synthetic sea surface elevations, Equation (11) was used in our study. The scale parameter *a* and the translation parameter *b* in this formula was set up based on the terms *t* and *ω* from *W_η_*(*t,ω*). The Morlet wavelet function was still implemented in Equation (11). Figure 4 presents a comparison of one set of observational sea surface elevations *η*′(*t*) with its synthetic sea surface elevations *η*(*t*). The normalised differences *N_d_* between *η*′(*t*) and *η*(*t*) is defined as:

(15) $$N_{d}(t)=\frac{\eta (t)-\eta^{\prime }(t)}{H_{s}}$$

in which *H_s_* is the significant wave height estimated from the observational wave records by the zero-up-crossing method [24]. In this analysis, we used the identical wave case as presented in Figure 2. The synthetic sea surface elevations are closed to the observational sea surface elevations, except for the results at the beginning and the end of the time series. For the normalised differences between the synthetic sea surface elevations and the observational sea surface elevations within the middle part of the time series (*t* = 129∼384 s), the mean value of absolute differences is 0.067, and the standard deviation of is 0.085. However, the differences at the two ends of the time series can be up to three times of the amplitude of the original data. The inaccurate results at the two ends of the time series are due to signal leakage. To verify the influence of leakage on synthesizing sea surface elevations, an extensive discussion could be found below.

## 4. Discussion

### 4.1. The Ideal Cut-Off Frequency

**Figure 5.**
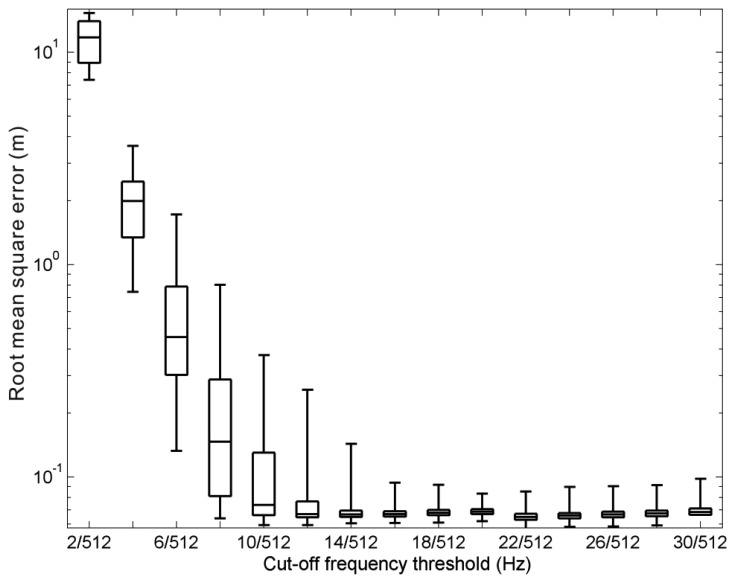
Simulation errors of sea surface elevations for different cut-off frequencies. The box-whisker plot shows that the estimated results are more accurate if the cut-off frequency is larger than 16/512 Hz.

The obvious normalised differences shown at very-low-frequency bins indicate that a high-pass filter is necessary to eliminate the noises caused by the effect of transfer function *ω*^−4^ when we apply Equation (13) to wave signals. This section will discuss the ideal cut-off frequency of high-pass filter. The same 1,500 cases used previously were also used to examine the effects of different cut-off frequency values on the wavelet spectrum filter. Because the sampling rate of our wave records was 2 Hz, the frequency range of the wavelet spectrum was distributed from 0 Hz to 1 Hz, based on the Nyquist–Shannon sampling theorem. For each wave case measured from the pile station, 1,024 sample points were chosen to compare with their corresponding synthesized values of sea surface elevations. Because the wave record was discrete, the signals in the spectral domain were discretised into 512 bins from 0 Hz to 1 Hz, while the other 512 bins were distributed from −1 Hz to 0 Hz. This established cut-off frequency values from 2/512 Hz to 30/512 Hz. Note that 30/512 Hz is greater than 0.05 Hz, which was originally set as the cut-off frequency for the case in Figure 2. Figure 5 shows the root mean square error (*R_e_*) between the 1,500 sea surface elevation records and the corresponding synthetic sea surface elevations for different cut-off frequency conditions:

(16) $$R_{e}=\sqrt{\overset{k}{\underset{t=1}{\text{∑}}}{\left[\eta (t)-\eta^{'}(t)\right]}^{2}/k}$$

in which *η*(*t*) is the synthetic sea surface elevations, *η*′(*t*) is the observational sea surface elevations, *k* is the number of total samples for each wave record. In our study, *k* equals to 1,024.

The results of this analysis indicated that to obtain an accurate estimate of the sea surface elevations from the wavelet spectrum, the cut-off frequency should not be less than 16/512 Hz. Finally 22/512 Hz was chosen as the cut-off frequency for our following treatments because its corresponding root-mean-square-error of the 75th percentile of data set is lowermost in Figure 5.

### 4.2. Ideal Margin Width of Wave Signals

Figure 2 has shown prominent inaccuracies on estimating the wavelet spectrum of sea surface elevations at the beginning and the end of the time period considered. These inaccurate estimations occur because of the limited expansion length of the wavelet function that generates the spectral leakage. The wavelet function is not complete at the locations of interest near the marginal region of the signal because its energy distribution is cut off at the two ends of the signal. After applying this incomplete wavelet function to the wave signal, the spectral energy leaks. However, the wavelets with larger scale parameters are always used to analyse the longer waves. This finding means the spectral leakage at the two ends of the signals would be more obvious when we analyse longer waves.

To evaluate the width of the marginal area, we need to consider the windows of the wavelet functions that indicate the effective zone of the wavelet functions. Outside this window, the amplitude of the wavelet function is weak enough to be neglected. In this analysis, we define a window that stops at the positions given by −*T* and *T* for the wavelet function. Jordan *et al.* [23] suggested a value of 3*σ_t_* for *T*, where the centre (first moment) *t*_0_ and the standard deviation (second moment) *σ_t_* of the wavelet function are defined as in Equations (17) and (18):

(17) $$t_{0}=\frac{{\int_{-\infty }^{\infty }t|\psi (t)|}^{2}dt}{{\int_{-\infty }^{\infty }|\psi (t)|}^{2}dt}$$

(18) $$\sigma_{t}={\left(\int_{-\infty }^{\infty }{\left(t-t_{0}\right)}^{2}{\left|\psi (t)\right|}^{2}dt\right)}^{0.5}$$

The |*ψ*(*t*)|^2^ of the Morlet mother wavelet can be simplified based on the Pythagorean identity:

(19) $${\left|\psi (t)\right|}^{2}=\exp(-t^{2})$$

Equation (19) shows that |*ψ*(*t*)|^2^ is an even function. In other words, the *t*_0_ value of the Morlet mother wavelet is equal to 0 and the *t*_0_ value of the Morlet wavelet function only depends on the translation parameter *b*. Using some known integration equations, we can derive the analytic equation for standard deviation of Morlet wavelet under the influences of scale parameter (*a*):

(20) $$\sigma_{t}={\left(0.5{\left(a^{6}\pi \right)}^{0.5}\right)}^{0.5}$$

Equation (20) reveals that the value of standard deviation is proportional to *a*^3/2^. Combining Equations (8), (17) and (18), we obtain the windows of the wavelet functions for different frequency conditions. Note that the frequency condition is related to the scale parameter *a* based on Equation (10). Equation (20) presents the analytic equation for standard deviation. To calculate the centre and the standard deviation of the discrete wavelet function, we must consider the number of total samples (*k*) and the sampling interval (Δ*t*):

(21) $$t_{0}=\frac{\overset{k}{\underset{t=1}{\text{∑}}}t{\left|\psi (t)\right|}^{2}\Delta t}{\overset{k}{\underset{t=1}{\text{∑}}}{\left|\psi (t)\right|}^{2}\Delta t}$$

(22) $$\sigma_{t}={\left(\overset{k}{\underset{t=1}{\text{∑}}}{\left(t-t_{0}\right)}^{2}{\left|\psi (t)\right|}^{2}\Delta t\right)}^{0.5}$$

**Figure 6.**
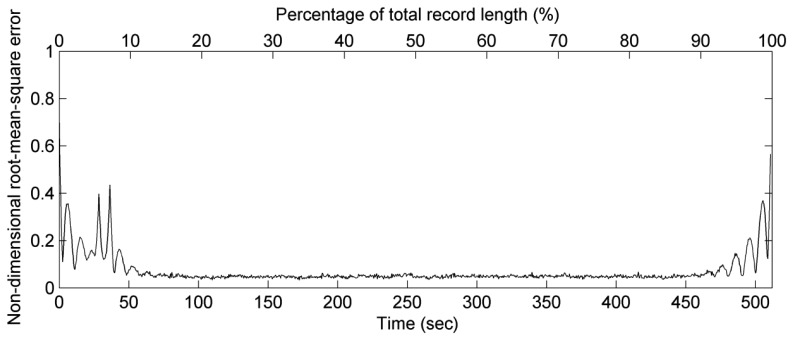
The non-dimensional root-mean-square errors between observational sea surface elevations and synthetic sea surface elevations from 1,500 wave cases. The errors are obvious and unstable at initial and final 70 s durations of a wave record.

In addition to the wavelet spectrum, the influence of signal leakage on synthesizing the sea surface elevations should be considered, as well. The width of the marginal area is also related to the wave frequency. According to wave theory, the sea surface elevations of irregular waves can be constructed by adding a large number of sinusoidal waves (component waves) with different amplitudes, frequencies, and phases [24]. To present all of the results from 1,500 different wave cases, we calculated the non-dimensional root mean square error *R_n_* between observational sea surface elevations *η*′(*t*) and synthetic sea surface elevations *η*(*t*):

(23) $$R_{n}(t)=\sqrt{\overset{N}{\underset{j=1}{\text{∑}}}{\left[\frac{\eta (t)-\eta^{\prime }(t)}{H_{sj}}\right]}^{2}/N}$$

in which *H_sj_* is the significant wave height of wave case *j*, *N* is the total number of wave cases. Figure 6 shows that the value of root mean square error is small and stable except when it is in a short period of 70 s from the edges of wave record.

The maximum wave period of our wave datasets is approximately 10 s. The window width of wavelet functions (3*σ_t_*) could then be estimated to be 72.9 s when we applied 10 s as the wave period condition to Equations (8), (10), (20) and (21). This estimated result for the window of wavelet function is quite similar to the result shown in Figure 6. It means that three times of the standard deviation is a proper threshold for the margin widths of estimated wavelet spectrum and synthetic sea elevations. Based on this result, the total marginal width is approximately 30% of the measured accelerations for a recording duration of 10 minutes in a wave buoy. A longer recording time can be used to reduce the percentage of the marginal width.

## 5. Wavelet Analysis of Acceleration Signals for the *in Situ* Wave Buoy

**Figure 7.**
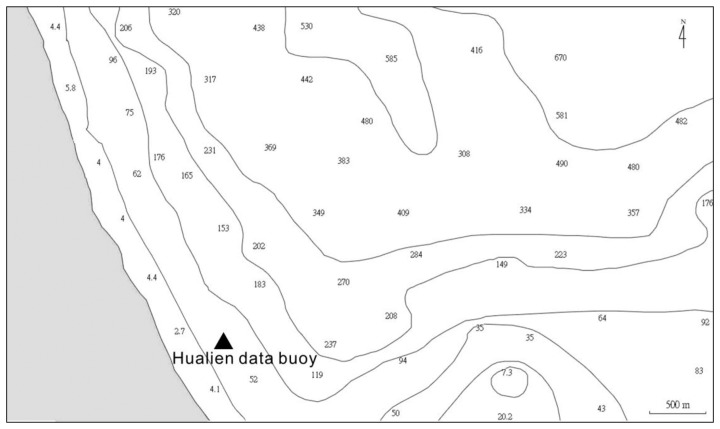
The location and surrounding bathymetry of buoy station. The numbers inside the figure stand for water depths. The water depth of buoy location is 30 m.

After confirming the feasibility of applying the aforementioned wavelet-based algorithm to observational wave records of a pile station, Equation (13) can now be used to synthesize sea surface elevations from the observational acceleration signals of a wave buoy. As shown in Figure 1, the data buoy of 2.5 m in diameter is located 0.4 km from the coast of eastern Taiwan (see Figure 1 for location), where the water depth is 30 m and surrounding bathymetry is shown in Figure 7. A total of 8,000 time-series records of acceleration signals were collected from the data buoy. Their significant wave heights and the mean wave periods are calculated from wave spectrum [24] and have a range of 0.5–3 m and 4–8 s, respectively. These results indicate that part of our data belongs to the cases of transitional water waves, which interact with the sea bed. Compared to the pile station, which is located inside the Taiwan Strait of finite water depth, the buoy is located in the open ocean (the Pacific). Thus, the wave heights and periods recorded from the buoy are often larger than those recorded from the pile station.

**Figure 8.**
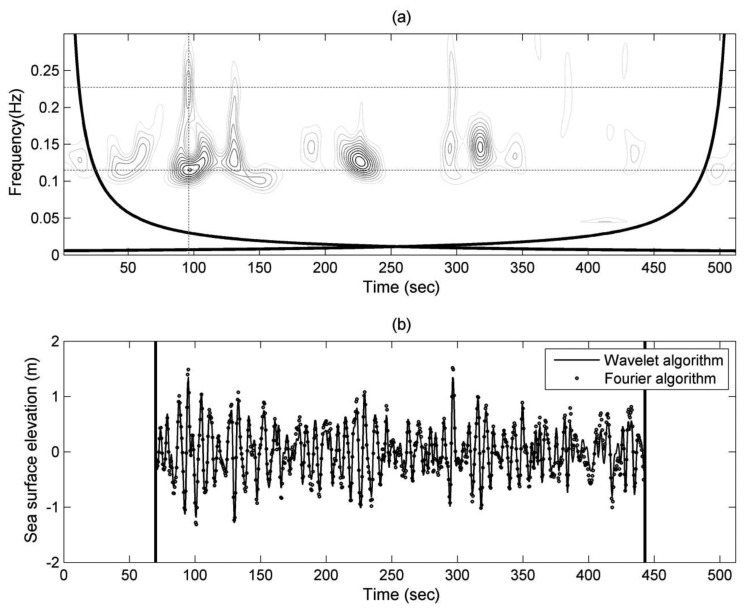
(**a**) A Wavelet spectrum of sea surface elevations; (**b**) Sea surface elevations derived from acceleration signals of wave buoy. The significant wave height and mean wave period of this record are 1.6 m and 5.8 s, respectively. During the period of between 90 s and 100 s of wavelet spectrum, the highest and 2nd highest peaks of energy distribution are located at around 0.115 Hz and 0.23 Hz, respectively. The horizontal dash lines in Figure (a) indicate the corresponding frequencies of two peaks.

One record of observational accelerations was chose to derive the wavelet spectrum of sea surface elevations (as shown in Figure 8a) with Equation (13), and its synthetic sea surface elevations (as shown in Figure 8b) could then be obtained by Equation (11). The ideal cut-off frequency discussed in Section 4.1 was applied to the calculation of wavelet analysis algorithm. To avoid inaccurate results because of the spectral leakage, we follow the suggestions of Section 4.2 and removed the results near the two ends of the wavelet spectrum and the synthesized time series of sea surface elevations. The synthetic sea surface elevations estimated by the wavelet-based algorithm are compared to the sea surface elevations calculated by the Fourier transform in Figure 8b. The comparison proves the feasibility of wavelet-based algorithm on synthesizing the sea surface elevations from observational accelerations.

The energy distribution of Figure 8a shows a noticeable existence of energy during the period between 90 s and 100 s. The highest peak of the energy distribution is located at around 0.115 Hz; the 2nd highest peak is located at around 0.23 Hz and twice the highest peak frequency. Some studies have reported the similar phenomenon which was found out from the wave spectrum. In examining the wave spectrum of an ocean wave time series, Herbich [30] observed that the secondary spectral peak at the frequency of approximately twice the main peak frequency is almost entirely composed of secondary nonlinear components that belong to the first group of bound waves. Nonlinear interactions can occur among waves with frequencies which satisfy the relationship [31]:

(24) $$f_{1}\pm f_{2}=f_{3}$$

where *f_i_* is the scalar frequency of the *i*-th wave component. One special case of this interaction condition is:

(25) $$f_{1}=f_{2}=f_{p}$$

where *f_p_* is the frequency of the spectral peak. Such self-interactions generate a harmonic of the spectral peak at 2*f_p_*. The magnitude of the second harmonic increased, and the waves were clearly observed to take a nonlinear shape. The natural waves in the ocean are often nonlinear, random and directionally spread. However, engineering calculations are typically performed using waves that are either linear and random or nonlinear and regular. The nonlinearity of ocean waves is often conspicuous in the shallow water of the coastal regions because of the influences of bathymetry [32,33]. However, some studies also observed the wave nonlinearity in deeper water depth [34]. To fully investigate the mechanics of natural waves, the wave nonlinearity in the intermediate or deep water should not be ignored.

**Figure 9.**
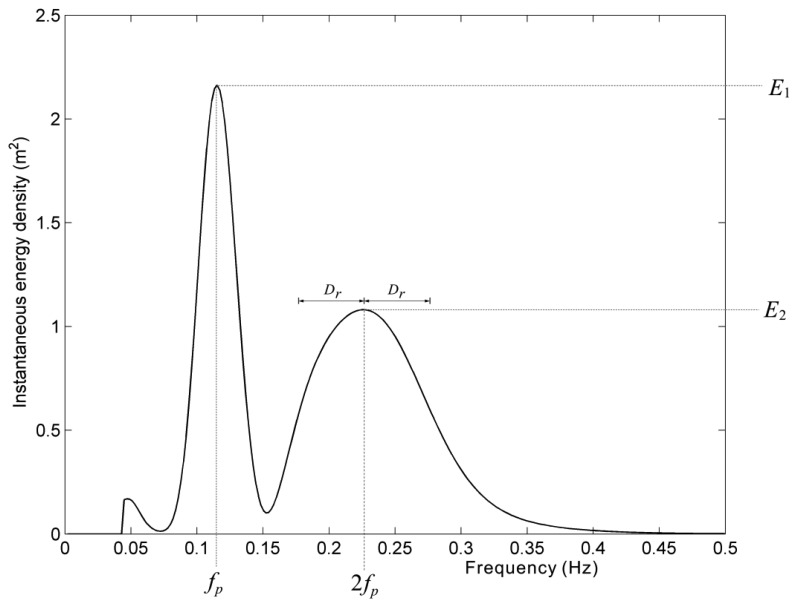
An instantaneous spectrum extracted from the wavelet spectrum of Figure 8a at 97 s.

To detect wave nonlinearity at the spectral peak of frequency 2*f_p_*, we need to determine the spectral peaks definitely. An instantaneous spectrum at 97 s was extracted from the wavelet spectrum of Figure 8a. The frequency bin of maximum energy density is defined as the major peak frequency *f_p_*. At the frequency of 2*f_p_*, we need to determine whether energy density peak exists or not. As shown in Figure 9, the energy density at 2*f_p_* must be the local highest one within the range of [2*f_p_*-*D_r_*,2*f_p_*+*D_r_*] if the spectral peak at 2*f_p_* exists.

**Figure 10.**
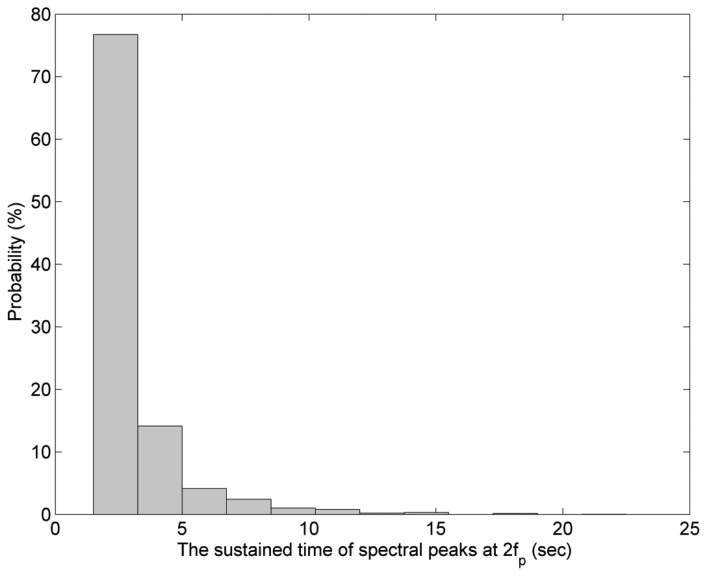
Probabilities of sustained time durations of the spectral peak at 2*f_p_* from 8,000 wavelet spectra.

To avoid the overlap of energy density between *f_p_* and 2*f_p_*, the value of *D_r_* was set up as 0.05 Hz in our study. Under this definition, we can observe an obvious spectral peak at 2*f_p_* from the wavelet spectrum. However, the spectral peak at 2*f_p_* is only sustained for several seconds. Figure 10 presents the probabilities of sustained time durations of the spectral peak at 2*f_p_* from the wavelet spectra of all wave records. However, the cases of duration shorter than 1 s were not included in Figure 10, because the sampling interval of wave records is 0.5 s. It shows that most of the sustained time durations are shorter than 5 s, *i.e.*, the existences of most of harmonic spectral peaks are transient. In other words, the phenomenon of wave nonlinearity can be non-stationary. Under this situation, the wave spectrum derived by Fourier transform isn't able to present the short-time wave features. Neither is the time-averaged wavelet spectrum.

**Figure 11.**
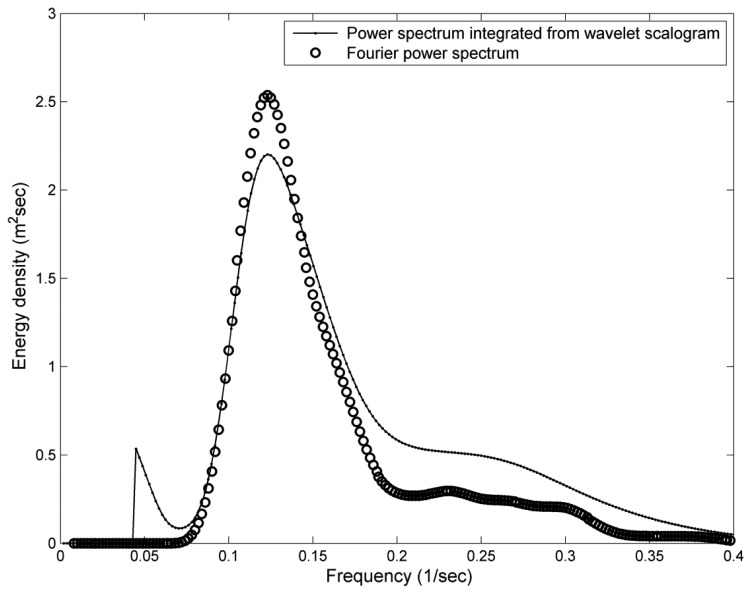
A comparison between a wave spectrum derived by Fourier transform and a time-averaged wavelet spectrum derived by wavelet transform.

A time-averaged wavelet spectrum, estimated by integrating the time domain of wavelet spectrum in Figure 8a, was compared to the wave spectrum, derived by Fourier transform from the same observational acceleration record, in Figure 11. To smooth the raw wave spectrum, a Hamming window of window length = 2.5 s was applied to the Fourier transform of the acceleration signals. The differences in energy density between the wavelet-based result and the smoothed Fourier power spectrum are expectable. One of the reasons is the power leakage of the wavelet spectrum. Compared to the Fourier transform, the time-averaged wavelet spectrum with power leaking into adjacent harmonics is unavoidable. Nevertheless, these two spectra are similar. The fundamental frequencies *f_p_* from these two spectra are both approximately 0.115 Hz. However, the spectral energy at 2*f_p_* (0.23 Hz) is not obvious from these two spectra as shown in Figure 11. Because the energy at different harmonic frequency bins is averaged in the wave spectrum, it is difficult to detect the wave nonlinearity features from a wave spectrum that is averaged over the entire 512 s time series. From 8,000 different acceleration records at our study site, 1,627 records show short-time nonlinear wave events. It means the occurrence of short-time nonlinear wave event can be up to 20%, even though the duration of this nonlinear wave event is only several seconds within the entire 512-s time series. Compare to the short-time nonlinear wave events, only 16 Fourier wave spectra show a harmonic of the spectral peak at 2*f_p_*. By means of wavelet spectrum, we have more chances to explore the nonlinear wave features which were hidden in the averaged spectrum.

After finding the nonlinear features that existed in a wavelet spectrum like the one shown in Figure 8a, we could also examine its corresponding short-time series of sea surface elevations. The short-time wave profile was derived from the wavelet algorithm and like the one shown in Figure 8b, but it only has short-time duration and concentrates at the instant when nonlinearity occurs. Firstly, the instantaneous spectrum like Figure 9 was extracted from the wavelet spectrum at the time of one major spectral peak at *f_p_* and the other minor peak at 2*f_p_*. Since the wave nonlinearity is affected by the energy density at 2*f_p_*, the ratio *R_f_* of the energy density *E*_2_ (as shown in Figure 9) at 2*f_p_* to the energy density *E*_1_ (as shown in Figure 9) at *f_p_* was used here to quantify the wave nonlinearity:

(26) $$R_{f}=E_{2}/E_{1}$$

In theory, the two components from *f_p_* and 2*f_p_* would reinforce each other at the wave crest of elevation profile and weaken each other at the wave trough. This phenomenon should yield a surface elevation profile of vertical asymmetry. To reveal the feature of short-time nonlinear event from the wave profile, the local wave vertical asymmetry *V_a_* [35] was applied in our succeeding discussion:

(27) $$V_{a}=A_{n}/H_{n}$$

where *A_n_* and *H_n_* are the amplitude and wave height, derived by the zero-up-crossing method [24], of short-time wave profile, respectively. The wave asymmetry has been used to discuss the wave instability and breaking in some studies.

**Figure 12.**
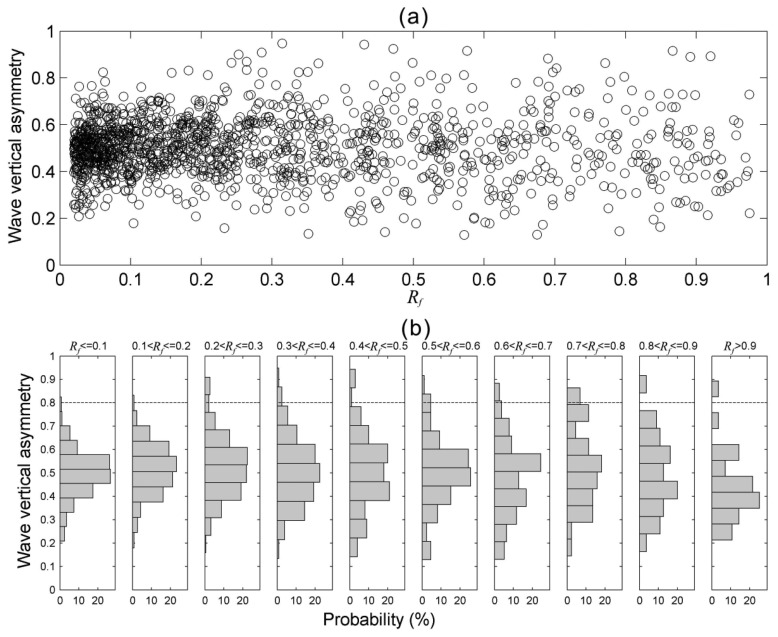
(**a**) A scatterplot of *R_f_* and *V_a_*; (**b**) A histogram of *V_a_* under different *R_f_*. The parameter *R_f_* is defined as the ratio of the energy density at 2*f_p_* to the energy density at *f_p_*. *V_a_* is defined as the ratio of amplitude to wave height of a short-time nonlinear wave profile.

After reviewing 8,000 wavelet spectra derived from *in situ* acceleration signals of the wave buoy, the relationship between *R_f_* and *V_a_* was acquired and is shown in Figure 12. It shows that most of the *V_a_* are in the range of value 0.3–0.7. However, more wave profiles with higher *V_a_* values were observed in the cases of *R_f_* > 0.2. In some cases *V_a_* can be even larger than 0.8. Some experiments, based on regular wave cases, showed the vertical asymmetry continuously increased as the wave shoaled and reached a maximum of between 0.62 and 0.74 at breaking [35]. However, real ocean waves in Nature are always irregular, random, and even nonlinear in some situations. Although the vertical asymmetry of a wave profile with higher crest and shallower trough could be estimated by means of zero-up-crossing method, its energy interaction between frequencies is hardly to inspect because most of harmonic spectral peaks are transient as mentioned and proved in the preceding paragraph. In this study the wave cases with very high vertical asymmetry values of *V_a_* > 0.8, their *R_f_* values was found to be larger than 0.2. This result implies that the wave instability should be related to short-time wave nonlinearity. To explore more detailed characteristics of wave nonlinearity, not only should the sea surface elevation be examined, but also its wavelet spectrum is worthy of being explored.

## 6. Conclusions

Accelerometers, which are commonly used in the airline industry, are also among the most useful sensors for ocean wave measurements. So far, the accelerometer-equipped buoy is one of the most popular tools to obtain wave information. The FFT-based algorithm is undoubtedly a suitable method to derive the wave spectrum and also sea surface elevations from buoy acceleration signals, however, the non-stationary or nonlinear phenomenon of wave energy may be concealed in the wave spectrum derived by Fourier transform. Hence a wavelet-based algorithm was developed to examine the local characteristics of wave nonlinearity in both the time and frequency domains. Although a large number of studies have applied the wavelet-based algorithm to examine the *in situ* wave records which were measured by wave gauges on offshore platforms, the issue of analysing the acceleration signals from a moored buoy has received little attention. Our objectives of the study are first to verify the accuracies of wavelet spectra and synthetic sea surface elevations which were derived by the wavelet-based algorithm from the observational acceleration signals of a wave buoy, and then the instantaneous wavelet spectrum and short-time wave profile of sea surface elevations could be extracted to quantify the wave nonlinearity and wave vertical asymmetry, respectively, at the instant when nonlinearity occurs in order to examine the relationship between wave instability and wave nonlinearity.

To accurately synthesize the sea surface elevations from the wavelet spectrum, it is necessary to reduce the low-frequency noises caused by the transfer function which transforms the wavelet spectrum of accelerations to a wavelet spectrum of sea surface elevations. A high-pass filter with a rectangular window was introduced to diminish the evident noises at very low frequencies. The ideal cut-off frequency of high-pass filter was then examined and determined for our cases. We also verified an ideal margin width of synthetic wave signals because the effect of spectral leakage always causes inaccurate estimations at the beginning and the end of time.

After confirming the feasibility of the wavelet-based algorithm, we applied the algorithm to synthesizing wavelet spectra of sea surface elevations from the observational acceleration signals of a wave buoy. The wavelet spectra showed that individual nonlinear wave features are instantaneous or short-time events. Most of these short-time nonlinear wave events occur for only several seconds. This suggests that the energies at different harmonic frequency bins might have been averaged in the Fourier-type wave spectrum. It also explains why the energy at the harmonic frequency is not noticeable in the wave spectrum, although it is apparent in the wavelet spectrum. In addition to the nonlinear features that are extracted from the wavelet spectrum, the wave profiles of individual nonlinear wave events were also examined in this study. By analysing the short-time wave profiles of sea surface elevation records, which were derived from acceleration signals of a wave buoy by the wavelet-based algorithm, we confirm the local wave vertical asymmetry is related to short-time wave nonlinearity. Hence, the wavelet spectrum and sea surface elevations are both significant to explore the short-time wave nonlinearity.

In previous studies on analysing nonlinear wave data, most observational data are sea surface elevation records and were measured in shallow waters because nonlinear wave phenomena are common and easily observed there. However, nonlinearity can also occur in waters of deep or intermediate depth, where *in situ* wave data are mainly recorded by wave buoys. The original wave signals observed by a buoy are wave accelerations and are seldom directly used to discuss the nonlinearity of water waves. Our study confirmed that the wavelet-based algorithm is practical for extracting the short-time nonstationary and even nonlinear information from the buoy acceleration records, so the algorithm will provide us a new tool to explore more wave features in deep and intermediate waters. In summary, this study confirms the feasibility and manifests the benefits of wavelet-based algorithm on analysing wave acceleration signals.

## List of Symbols

*a*: Scale parameter
*b*: Translation parameter
*A_c_*: Acceleration signal
*A_n_*: Amplitude of nonlinear wave profile
*C_ψ_*: Admissibility constant
*C_c_*: Correlation coefficient of the wavelet spectra
*E*_1_: Energy density at major peak frequency of wave spectrum
*E*_2_: Energy density at 2*f_p_*
*E_n_*: Total energy of signal
*f_i_*: Scalar frequency of the *i*-th wave component
*f_p_*: Major peak Frequency of wave spectrum
*g*: Gravitational acceleration
*H_n_*: Wave height of nonlinear wave profile
*H_s_*: Significant wave height synthesized from observational wave records by the zero-up-crossing method
*H_sj_*: The significant wave height of wave case *j*
*k*: Number of total samples number of total samples for each wave record
*N*: Total number of wave cases
*N_d_*: Normalised differences between *η*′(*t*) and *η*(*t*)
*R_e_*: Root mean square error
*R_n_*: Non-dimensional root mean square error
*R_f_*: Ratio of the energy density at 2*f_p_* to the energy density at *f_p_*
*t*_0_: Centre of the wavelet function
*V_a_*: Local wave vertical asymmetry
|*W_AC_*(*b,a*)|^2^: Scalogram of acceleration signa
|*W_AC_*(*t,ω*)|^2^: Wavelet spectrum of acceleration signal
|*W_η_*(*b,a*)|^2^: Scalogram of sea surface elevations
|*W_η_*(*t,ω*)|^2^: Wavelet spectrum of sea surface elevations
|*x*(*t,ω*)|^2^: Wavelet spectra of observational sea surface elevation data
|*y*(*t,ω*)|^2^: Estimated Wavelet spectra of sea surface elevation data, which are derived from synthetic acceleration data
*ψ_b,a_*: Transformed wavelet function
*ψ*_b,a_*: The complex conjugate of wavelet function
*ψ*: Mother wavelet function
*ψ′*: Fourier space of *ψ*
*ω*_0_: A constant that forces the admissibility condition
*ω*: Angular frequency
*μ_x_*: Mean values of |*x*(*t,ω*)|^2^
*μ_y_*: Mean values of |*y*(*t,ω*)|^2^
*σ_t_*: Standard deviation of wavelet function
*σ_x_*: Standard deviations of |*x*(*t,ω*)|^2^
*σ_y_*: Standard deviations of |*y*(*t,ω*)|^2^
*η*(*t*): Synthetic sea surface elevations
*η*′(*t*): Observational sea surface elevations
Δ*t*: Sampling interval of the signals
